# Early Intestinal Ultrasound Assessment Predicts Therapy Response: An Easy Tool for Clinical Decision-Making

**DOI:** 10.1093/ibd/izaf317

**Published:** 2026-03-06

**Authors:** Elena De Cristofaro, Francesca Zorzi, Alice Colella, Luca Basile, Fabiana Castiglione, Antonio Rispo, Anna Testa, Alessia Dalila Guarino, Elisabetta Lolli, Elisa Cuccagna, Giovanni Monteleone, Emma Calabrese

**Affiliations:** Gastroenterology Unit, Department of Systems Medicine, University of Rome Tor Vergata, Rome, Italy; Gastroenterology Unit, Department of Systems Medicine, University of Rome Tor Vergata, Rome, Italy; Gastroenterology Unit, Department of Systems Medicine, University of Rome Tor Vergata, Rome, Italy; Gastroenterology Unit, Department of Systems Medicine, University of Rome Tor Vergata, Rome, Italy; Gastroenterology, Department of Clinical Medicine and Surgery, University of Federico II, Naples, Italy; Gastroenterology, Department of Clinical Medicine and Surgery, University of Federico II, Naples, Italy; Gastroenterology, Department of Clinical Medicine and Surgery, University of Federico II, Naples, Italy; Gastroenterology, Department of Clinical Medicine and Surgery, University of Federico II, Naples, Italy; Gastroenterology Unit, Department of Systems Medicine, University of Rome Tor Vergata, Rome, Italy; Gastroenterology Unit, Department of Systems Medicine, University of Rome Tor Vergata, Rome, Italy; Gastroenterology Unit, Department of Systems Medicine, University of Rome Tor Vergata, Rome, Italy; Gastroenterology Unit, Department of Systems Medicine, University of Rome Tor Vergata, Rome, Italy

**Keywords:** intestinal ultrasound, transmural healing, Crohn’s disease, monitoring treatment

## Abstract

**Background:**

Transmural healing (TH) has emerged as a therapeutic target in Crohn’s disease (CD), providing a more comprehensive indicator of deep remission than mucosal healing alone. Intestinal ultrasound (IUS) is a noninvasive method for assessing TH, but its prognostic value remains insufficiently defined.

**Objective:**

The aim of this prospective study was to evaluate whether early improvement in IUS parameters during biological therapy could predict TH at 12 months.

**Design:**

This is a prospective multicenter study enrolling CD patients initiating biological therapies. IUS and Doppler parameters were assessed at baseline, 3 months, and 12 months. Delta (Δ) represented the variation in ultrasound measurements between baseline and 3 months. TH was defined as normalization of bowel wall features and absence of hypervascularization.

**Results:**

A total of 142 CD patients were included. At 12 months, the TH rate was 19%, the IUS response rate was 44%. Patients achieving TH showed a significantly greater ΔBWT than nonresponders (*P* = .0004). On ROC analysis, a ΔBWT reduction of 1.25 mm predicted TH with 73% sensitivity and 61% specificity. IUS responders had a significantly greater ΔBWT than nonresponders (*P* < .0001), with the same threshold predicting response with 83% sensitivity and 57% specificity. Notably, the combination of ΔBWT and Limberg score improvement was strongly associated with both TH (OR 13.26; *P* < .0001) and IUS response (OR 20.9; *P* < .0001) at 12 months.

**Conclusion:**

Early reduction in BWT, especially when combined with Limberg score, is a strong predictor of TH and IUS response at 12 months, supporting the use of early IUS monitoring in clinical practice.

Key Messages

- **What is already known on this topic**
  - *Transmural healing (TH) has emerged as a treatment target beyond mucosal healing in Crohn’s disease (CD), and is associated with improved long-term outcome Intestinal ultrasound (IUS) is a noninvasive tool increasingly used for monitoring disease activity and treatment response, but its role in early prediction of TH remains underexplored.*
- ***What this study adds***
  - *Early changes in bowel wall thickness (ΔBWT) and Limberg score at 3 months predict TH at 12 months.*
  - *A ΔBWT >1.25 mm and a combined improvement in BWT and Limberg score are strongly associated with TH and IUS response.*
- ***How this study might affect research, practice or policy***
  - *IUS can support early treatment decisions in a tight monitoring strategy, serving as a noninvasive tool to predict deep healing and guide therapy optimization.*

## Introduction

Crohn’s disease (CD) is a chronic, idiopathic, and disabling inflammatory disorder characterized by persistent transmural inflammation, leading to progressive structural bowel damage and intestinal complications such as strictures, fistulae, and abscesses. Despite advancements in disease management, achieving long-term remission remains a challenge, requiring accurate and timely assessment of treatment response. Historically, mucosal healing (MH) has been considered a key therapeutic target in CD.^1^ However, MH primarily reflects superficial mucosal changes and does not capture transmural inflammation, which plays a crucial role in disease progression. As a result, MH alone fails to fully represent the disease burden or predict long-term outcomes, highlighting the need for alternative assessment tools. In this context, intestinal ultrasound (IUS) is a valuable, noninvasive technique for evaluating transmural healing (TH).^2^ TH is increasingly recognized as a more comprehensive goal in CD patients receiving biological or small molecules therapy, as it better reflects deep tissue healing and long-term disease control.^3^^,^^4^ In a previous multicenter study, we confirmed that IUS is a reliable and easy to use tool for monitoring biologic-induced improvements in bowel inflammation in CD.^5^ Several ultrasound-based activity scores have been developed to assist in managing patients with CD.^6–8^ However, the responsiveness in terms of ability to detect clinically relevant sonographic changes over time, has been evaluated for only a limited number of these scores.^9^ Different studies have demonstrated that normalization of bowel wall thickening (BWT), or a positive IUS response is associated with improved long-term outcomes.^5^^,^^10^ TH is associated with a higher rate of steroid‐free clinical remission and a lower rate of clinical relapse at 1‐year compared to mucosal healing and no healing.^3^ TH is also associated with significantly lower hospitalization rates, than mucosal healing and no indication for surgery was found in cases of TH.^3^ The same outcomes were analyzed and confirmed in a subsequent study with a median long term follow up of 3 years.^11^ However, data on the prognostic value of IUS in early predicting the achievement of TH after biologic therapies remain limited. Thus, the aim of our study was to evaluate whether early IUS-detected lesion improvement during biological therapy could predict the achievement of TH at 12 months.

## Methods

### Study Population and Study Design

This prospective observational study was conducted at two inflammatory bowel disease (IBD) tertiary centers: the University of Rome Tor Vergata and the Federico II University Medical School in Naples. We enrolled adult patients with a confirmed diagnosis of ileal and/or ileocolonic CD who had an indication for biological therapy, including infliximab, adalimumab, vedolizumab, ustekinumab, or risankizumab. Diagnosis, disease location, and clinical assessments were performed according to internationally accepted recommendations. Patients were eligible for inclusion if they were over 18-year old and required biologic therapy for active luminal disease defined by evidence of activity on endoscopy (SES-CD > 3), IUS (bowel wall thickness [BWT] > 3 mm), and clinical assessment, regardless of prior intestinal surgery. Exclusion criteria included the presence of ileal or colonic stoma, obesity (BMI > 30), gastroduodenal or anorectal involvement, abdominal abscesses, or pregnancy. Each patient underwent IUS at baseline, at 3 months, and at 12 months following the initiation of biological therapy. Ultrasound features were prospectively collected and recorded at each point. The clinical activity was calculated using Harvey Bradshaw Index (HBI). The use of concomitant steroids at baseline was systematically recorded. Written informed consent was obtained from all study participants. The study was approved by the local ethics committee of the study coordinator center (number 174/17). All authors had access to the study data and reviewed and approved the final manuscript.

### Intestinal Ultrasound Parameters

IUS assessments were performed at baseline, 3 months, and 12 months using standard protocols. All patients were examined in the fasting state by experienced intestinal sonographers, utilizing a convex probe (1-8 MHz) and a high-frequency linear-array transducer (3-11 MHz).

The ultrasound parameters evaluated included BWT, defined as a thickening >3 mm at baseline measured in both longitudinal and transverse planes, lesion length (defined as the contiguous pathologically thickened bowel wall), echopattern (preserved or lost), and blood flow assessed using Limberg score.^12^ Complications were identified as the presence of stenosis (narrowed lumen < 10 mm, with (> 25 mm) or without dilation of a proximal loop), fistulas (defines as hypoechoic tracts with or without hyperechoic content), or fissures. The most affected bowel segment at baseline (dominant segment) was used for all IUS parameters and consistently evaluated throughout the study period. Additionally, fibrofatty proliferation and lymphadenopathies (short axis ≥ 5 mm) were recorded per patient basis.

At 3-month follow-up, IUS findings were categorized as follows:

1. TH: Defined as the complete normalization of all ultrasound parameters with a BWT < 3 mm
2. IUS responders: Differently from the IBUS consensus definition,^13^ we defined IUS responders as patients showing an improvement in BWT (>1 mm) along with a reduction in lesion length, Limberg score improvement, and no worsening of other parameters. All patients with improved lesions had at least 2 improved ultrasonographic parameters.
3. Nonresponders: Patients with either unchanged or worsened IUS findings.

To quantify ultrasonographic changes, a delta (Δ) value was calculated, representing the difference of BWT and Limberg score between baseline and 3-month. The achievement of TH at 12 months was subsequently assessed, and the Δ values were analyzed to determine their predictive value for transmural healing after one year.

### Biological Therapies

In this prospective study, patients received biologic treatment according to ECCO guidelines and standard clinical practice, including infliximab, adalimumab, vedolizumab, ustekinumab, and risankizumab. Induction and maintenance regimens followed approved dosing schedules, with reactive treatment optimization performed at the discretion of the treating physician when loss of clinical response was associated with objective evidence of active disease (elevated biomarkers and/or IUS activity). Treatment was intensified reactively when a loss of clinical response was documented, provided it was associated with objective evidence of active disease, as demonstrated by elevated fecal calprotectin or C-reactive protein, and confirmed by IUS findings. Decisions were made at the discretion of the treating physician, integrating all available clinical, biochemical, and sonographic data. Detailed dosing and administration protocols are provided in the Supplementary Materials (Table S1).

### Outcomes

The primary endpoint was to assess whether changes in BWT (Δ) at 3 months could predict TH at 12 months after initiating biological therapy.

Secondary endpoints included evaluating whether ΔBWT alone or in combination with the Limberg score at 3 months could predict IUS response at 12 months and identifying predictive factors of TH at 12 months.

### Statistical Analysis

Demographic data were presented as numbers and percentages for categorical variables, and as median and range for continuous variables. Changes in IUS parameters at 3 and 12 months were analyzed using the Kruskal–Wallis test. Correlations between changes in clinical indices and sonographic parameters were assessed using Spearman’s rank correlation coefficient. Differences between combinations of categorical variables were assessed using the Chi-square test. Logistic regression analysis was performed to evaluate the association between therapeutic outcome (TH) at 12 months (dependent variable) and potential predictors (independent variables). A *P*-value < .05 was considered statistically significant, and all variables with significant univariate associations were included in the multivariate model. Receiver Operating Characteristic (ROC) curve analysis was conducted to determine the optimal ΔBWT cut-off at 3 months predictive of TH and IUS response at 12 months. Analyses were performed in a modified intention-to-treat population; data from patients who discontinued treatment were considered missing.

## Results

### Study Population at Baseline

A total of 142 patients were included at baseline. Eighty-eight patients were male (62%), the median age was 39.5 years (range: 18-74 years), and the median disease duration was 96 months (range: 3-564 months). There was a homogeneous representation of different disease behaviors: 44 patients (31%) had a nonstricturing, nonpenetrating behavior (B1), 53 (37.3%) had a fibrostricturing behavior (B2), and 45 (31.7%) had a penetrating behavior (B3). The most prevalent disease locations were ileal (*n* = 70; 49.3%) and ileal-colonic (*n* = 59; 41.6%). A total of 61 patients (43%) had previously undergone CD-related surgery, while 78 (55%) had prior exposure to anti-TNFα therapies. At baseline, 34 patients (24%) were receiving steroid treatment.

The median clinical activity score, as measured by the HBI at baseline, was 6 (0-18). The prescribed biological therapies were: 16 patients (11.3%) started Infliximab, 70 (49.3%) adalimumab, 45 (31.7%) ustekinumab, 10 (7%) vedolizumab, and 1 (0.7%) risankizumab (Table 1).

**Table 1.** Characteristics of study population at baseline.

Characteristics	*n *= 142
**Sex, male, *n* (%)**	88 (62)
**Age, years, median (range)**	39.5 (18-74)
**CD duration, months, median (range)**	96 (3-564)
**Age at diagnosis, *n* (%)**	
-**A1**	17 (12)
-**A2**	103 (72.5)
-**A3**	22 (15.5)
**CD behavior, *n* (%)**	
-**B1**	44 (31)
-**B2**	53 (37.3)
-**B3**	45 (31.7)
**CD location, *n* (%)**	
-**L1**	70 (49.3)
-**L2**	13 (9.1)
-**L3**	59 (41.6)
**Perianal disease, *n* (%)**	29 (20.4)
**Prior CD related surgery, *n* (%)**	61 (43)
**Prior anti-TNFs, *n* (%)**	78 (55)
**Harvey Bradshaw Index at enrolment, median (range)**	6 (0-18)
**Biological therapy started, *n* (%)**	
-**Infliximab**	16 (11.3)
-**Adalimumab**	70 (49.3)
-**Ustekinumab**	45 (31.7)
-**Vedolizumab**	10 (7)
-**Risankizumab**	1 (0.7)
**Steroid treatment, *n* (%)**	34 (24)

Regarding IUS parameters, 115 patients (77%) had dominant ileal disease, while 34 patients (23%) had a dominant colonic disease location. The median BWT at baseline was 7 mm (4-12 mm), with a median lesion extent of 15 cm (5-60 cm). A total of 25 patients (18%) had a stenosis, while 15 patients (11%) had fistulas. The Limberg score was classified as absent or mild (Limberg score 1-2) in 64 patients (45%) and moderate to severe (Limberg score 3-4) in 78 patients (55%). The echopattern was preserved in 115 patients (81%) and lost in 27 (19%). Lymphadenopathy was observed in 58 patients (41%), while fibrofatty proliferation was detected in 96 (68%).

### Clinical and IUS Parameters over Time

After 3 months of biological therapy, 76 patients (53.5%) achieved clinical remission (HBI < 5), 50 patients (35.2%) had mild disease activity (HBI  ≥ 5 and < 7), and 16 patients (11.3%) experienced moderate activity (HBI  ≥ 8 and < 16). Additionally, nine patients (6.3%) required dose optimization, while 1 patient (0.7%) discontinued therapy due to adverse events. IUS response was observed in 33 (23%) patients (40 [28%] patients according to IBUS definitions; Table S2) and TH in 8 (5.6%). Six out of 8 patients (75%) with TH were also in clinical remission (HBI < 5). The agreement between clinical disease and IUS TH was 49.3% (*κ* = 0.05; Table S3).

After 12 months of biological therapy, 96 patients (67%) were in clinical remission (HBI < 5), 27 (19%) had mild activity (HBI  ≥ 5 and < 7), and 8 (6%) moderate activity (HBI  ≥ 8 and < 16). Fourteen patients (10%) needed optimizing therapy and 11 patients (8%) stopped therapy: two patients due to adverse events and nine patients to secondary failure.

Compared to baseline, a significant reduction of HBI score was observed after 3 and 12 months (*P* < .0001; Figure S1). Changes in HBI showed no significant correlation with changes in BWT (Spearman’s *ρ* = 0.07, 95% CI −0.11 to 0.23, *P* = .44). Similarly, at 12 months the correlation remained weak and not statistically significant (*ρ* = 0.16, 95% CI −0.02 to 0.33, *P* = .07)

Regarding IUS parameters, a significant reduction in BWT was observed at 12 months (5.6 mm vs 7.0 mm; *P <* .0001), along with improvements in lesion extent (ileal location: 11 cm vs 18 cm and colonic location 10 vs 30 cm; *P <* .000), presence and grade of vascularization (*P <* .0001). Additionally, lymphadenopathy and fibrofatty proliferation were observed in 27 patients (21%) and 62 patients (47%), respectively, both showing a significant reduction compared with baseline (*P = *.0004 and *P = *.0009, respectively). After 12 months of therapy, IUS response was observed in 62 (44%) patients [58 (41%) patients according to IBUS definitions; Table S2) and TH in 27 (19%). Twenty-four out of 27 patients (89%) with TH were in clinical remission (HBI <5) after 12 months. The agreement between clinical disease activity and TH was 42.7% (*κ* = 0.10; Table S3). The IUS findings at baseline, after 3 and 12 months of therapy are summarized in Table 2.

**Table 2.** Comparison of intestinal ultrasound characteristics (IUS) at baseline, at 3 and 12 months.

IUS characteristics	Baseline (142 pts)	After 3 months (141 pts)	After 12 months (131 pts)	*P* value
**BWT (mm)**				
**median (range)**	7 (4-12)	6 (3-12)	5.6 (2-12)	<0.0001
**Lesion extent (mm) median (range)**				
**Ileal disease**	18 (5-60)	15 (0-60)	11 (0-60)	<0.0001
**Colonic disease**	30 (10-60)	20 (0-60)	10 (0-60)	
**Stenosis, yes *n* (%)**	25 (18)	26 (18)	24 (18)	0.98
**Limberg score**				
**Absent/mild *n* (%)**	64 (45)	78 (55)	92 (70)	0**.0001**
**Moderate/severe *n* (%)**	78 (55%)	64 (45)	39 (30)	
**Echopattern**				
**Preserved *n* (%)**	115 (81)	121 (86)	114 (87)	0.227
**Lost *n* (%)**	27 (19)	20 (14)	17 (13)	
**Fistula, yes** ***n* (%)**	15 (11)	14 (10)	7 (5)	0.256
				

### IUS Changes (Δ) at 3 Months and Prediction of TH at 12 Months

**Figure 1.**
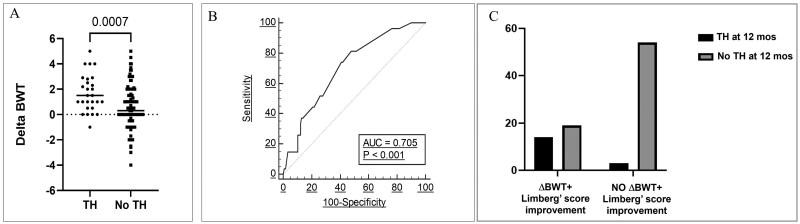
Patients achieving TH at 12 months had a higher ΔBWT than patient without TH (A). On ROC curve, ΔBWT improvement of 1.25 mm showed sensitivity and specificity of 73% and 61%, respectively, in predicting pts who achieved TH (B). A combination of ΔBWT (>1 mm) and Limberg score improvement (1 point) was associated with a higher rate of TH at 12 months with an OR of 13.26 (C).

The analysis of ΔBWT between baseline (T0) and 3 months (T3) showed an average change of 0.98 mm (±1.76 mm), ranging from −4 mm to + 5 mm. Regarding vascularization, ΔLimberg score at 3 months had an average value of 0.33 (±0.9). Patients who achieved TH at 12 months exhibited a significantly higher ΔBWT compared to those who did not (*P* = .0004; Figure 1A). ROC curve analysis identified a ΔBWT improvement of 1.25 mm as the optimal cut-off for predicting TH, with a sensitivity of 73% and a specificity of 61% (Figure 1B). The corresponding AUC was 0.705 (95% CI 0.62-0.78, *P* < .001).

The combination of ΔBWT >1 mm and an improvement in the Limberg score of 1 point was strongly associated with a higher likelihood of TH at 12 months (OR 13.26, 95% CI 3.59-45.64; *P* < .0001; Figure 1C).

### IUS Changes (Δ) at 3 Months and Prediction of IUS Response at 12 Months

**Figure 2.**
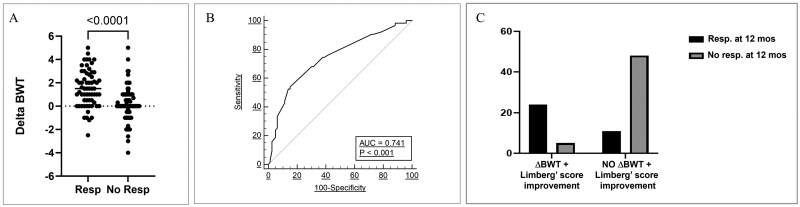
IUS responder group at 12 months had a higher ΔBWT than bowel U.S. nonresponder group (A). On ROC curve, ΔBWT improvement of 1.3 mm showed sensitivity and specificity of 52% and 86%, respectively, in predicting IUS responders (B). A combination of ΔBWT (≥1 mm) and Limberg score improvement (1 point) was associated with a higher rate of IUS responders at 12 months with an OR of 20.9 (C).

At 12 months, the IUS responder group had a significantly higher ΔBWT (1.57 ± 1.6 mm) compared to the IUS nonresponder group (0.18 ± 1.44 mm, *P* < .0001). Similarly, the ΔLimberg score improvement was greater in responders than in nonresponders (0.76 ± 1.0 vs -0.012 ± 0.62; *P* < .0001; Figure 2A). On the ROC curve, a ΔBWT improvement of 1.3 mm predicted IUS response with a sensitivity of 52% and a specificity of 86% (Figure 2B). The corresponding AUC was 0.741 (95% CI 0.66-0.811, *P* < .001).

**Figure 3.**
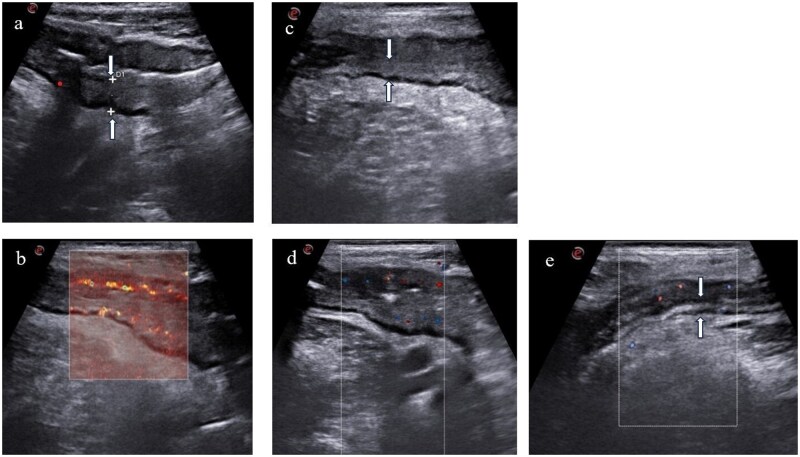
(A and B) Inflammation in the terminal ileum seen on intestinal ultrasound consistent with severely active Crohn’s disease and characterized by increased bowel wall thickness to 10 mm (white arrows, panel A) and severe grade of vascularization (Limberg score 4, panel B) before anti-TNF⍺ initiation. (C and D) After three months of biological therapy, improvement of bowel wall thickness of the terminal ileum (6 mm, white arrows, panel C) with mild grade of vascularization (Limberg score 2, panel D) (ΔBWT [4 mm] and Limberg score improvement [2 points] applied to this case). (E) At 12 months of biological therapy, further improvement of bowel wall thickness of the terminal ileum (4 mm, white arrows) with persistent mild grade of vascularization (Limberg score 2).

The combination of ΔBWT >1 mm and a 1-point improvement in the Limberg score was strongly associated with a higher likelihood of TH at 12 months (OR 20.9, 95% CI 6.41-52.4; *P* < .0001; Figures 2C and 3A-E).

### Overall Predictive Factors of TH at 12 Months

The predictive factors for TH were analyzed using a logistic regression model. Univariate analysis identified colonic disease location (OR 4.25, 95% CI 1.67-10.8; *P* = .002), a greater ΔBWT (OR 1.55, 95% CI 1.18-2.05; *P* = .001), and a higher ΔLimberg score (OR 2.52, 95% CI 1.52-4.17; *P* < .0001) as factors positively associated with TH at 12 months. Conversely, prior CD-related surgery (OR 0.31, 95% CI 0.12-0.83; *P* = .01), fibrostricturing behavior (OR 0.03, 95% CI 0.03-0.35; *P* = .0002), penetrating behavior (OR 0.13, 95% CI 0.04-0.42; *P* = .0007), and a greater baseline BWT (OR 0.65, 95% CI 0.47-0.89; *P* = .005) were inversely correlated with TH at 12 months.

The multivariate analysis confirmed colonic disease location (OR 8.93, 95% CI 1.77-45.11; *P* = .008), greater baseline BWT (OR 0.37, 95% CI 0.21-0.66; *P* = .0008), and greater ΔBWT (OR 2.37, 95% CI 1.38-4.11; *P* = .001) as independent predictive factors for TH (Table 3).

**Table 3.** Univariate and multivariate analyses to identify predictive factors of transmural healing (TH) at 12 months.

Predictors		Univariate			Multivariate		
		*P* value	OR	95% CI	*P* value	OR	95% CI
	Disease site evaluated at IUS. (ileal disease ref.)						
	- Colonic disease	0**.002**	**4.25**	**1.67-10.8**	**.008**	**8.93**	**1.77-45.1**
	Prior surgery (no ref.)	0**.01**	**0.31**	**0.12-0.83**	0.6	0.66	0.16-2.74
	Prior TNFs (no ref.)	0.22	0.59	0.25-1.38			
	Disease duration (mos)	0.08	0.99	0.99-1.01			
	Type of biological therapies (Ada ref.)						
	- Infliximab	0.42	0.52	0.11-2.56			
	- Vedolizumab	0.21	2.4	0.61-9.79			
	- Ustekinumab	0.28	0.56	0.2-1.58			
**TH** **At 12 months**	Presence of stenosis (no ref)	0**.01**	**0.14**	**0.01-1.14**	0.71	1.58	0.13-19.2
	Behavior (ref B1)						
	B2	0**.0002**	**0.11**	**0.03-0.35**	0.09	0.28	0.06-1.27
	B3	0**.0007**	**0.13**	**0.04-0.42**	0.33	0.44	0.08-2.35
	Age at enrolment	0.11	0.97	0.94-1.01			
	Echopattern	0.24	1.69	0.46-6.19			
	BWT (mm)	0**.005**	**0.65**	**0.47-0.89**	0**.0008**	**0.37**	**0.21-0.66**
	Fibrofatty proliferation	0.14	0.45	0.19-1.03			
	Limberg score (absence-mild vs moderate severe)	0.11	0.5	0.21-1.73			
	ΔBWT	0**.001**	**1.55**	**1.18-2.05**	0**.001**	**2.37**	**1.38-4.11**
	Limberg score improvement	0**.0001**	**2.52**	**1.52-4.17**	0.56	1.22	0.62-2.39

## Discussion

The risk of progressing bowel damage in CD supports the need to adopt different treatment targets and monitoring strategies in these patients, regardless of their class of therapy. The idea of TH is evident as supplementary target in the STRIDE-II consensus update on treat-to-target strategies.^14^ In CD, it is proposed that TH should be used as an adjunct to endoscopic remission and may represent a deeper level of healing. IUS does not use radiation ensuring a real-time point of care assessment of CD patients^15^ and monitoring after commencement of therapies. The effectiveness of IUS in monitoring treatment response in IBD patients has been demonstrated in large cohorts.^5^^,^^16^ Furthermore, the first interventional multicenter trial using IUS provided valuable prospective data on IUS outcomes in ustekinumab-treated CD patients.^17^

Observational studies have also suggested that ultrasound remission may be associated with better long-term outcomes.^3^^,^^11^^,^^18^ Castiglione and colleagues demonstrated that TH was associated with a higher rate of steroid‐free clinical remission and a lower rate of clinical relapse at 1‐year compared to mucosal healing and no healing at endoscopy.^3^ Transmural healing was also associated with significantly lower hospitalization rates, than mucosal healing and no indication for surgery was found in cases of TH.^3^ The same outcomes were analyzed and confirmed in our study with a median long term follow up of 3 years.^11^

The definition of treatment response varies significantly across studies, with both absolute and relative changes from baseline being considered. In various studies, a treatment response has been defined as BWT decrease ranging from 0.5 to 2.5 mm from baseline, or as around 25% percentage reduction. Response and remission criteria are influenced by the timing of assessments and the specific drugs used, factors that probably contribute to the variability in definitions of response to treatment between studies. Different ultrasound monitoring algorithms were proposed in adult and pediatric populations.^2^^,^^19^ Several ultrasonography-based activity scores have been developed to assist in managing patients with IBD.^6^^,^^8^ However, the ability to detect clinically significant changes over time has been evaluated for only a limited number of these scores. Among them, the IBUS-SAS^20^ and the BUSS^21^^,^^22^ have undergone responsiveness assessment.

The ultrasonographic response in the first 3 months regardless of therapy may represent a predictor of a favorable outcome. Our findings show that IUS changes as early as 3 months after therapy initiation -particularly reductions in BWT and Limberg score- can predict both TH and IUS response at 12 months. A ΔBWT >1.25 mm was associated with TH (sensitivity 73%, specificity 61%), and similar threshold predicted IUS responders. Moreover, combining a ΔBWT >1 mm with a 1-point Limberg score improvement strongly correlated with TH and IUS response (OR 13.3 and 20.9, respectively). In the multivariable analysis, ΔBWT remained an independent predictor of TH along with disease location. The reason for the differing response between the ileum and colonic segments under biologic therapy remains unclear. A slower normalization of sonographic parameters such as BWT, in the ileum compared to the colon is consistent with previous findings.^5^^,^^16^ Anatomical factors, including a higher density of Peyer’s patches and distinct microbial colonization at the terminal ileum, may partially explain this variability in response.

These results suggest that early assessment of BWT and vascularization using IUS may serve as a simple and reliable tool to identify patients more likely to achieve deep healing, supporting timely optimization or switching of therapies in a tight monitoring strategy.

This study has several notable strengths, including its prospective, real-world design conducted across two expert IBD centers with experienced sonographers. Although the absence of a gold standard may be viewed as a limitation, the anatomical location and extent of lesions were well known in our cohort. Another potential limitation is the known interobserver and inter-equipment variability associated with IUS. However, the reproducibility of BWT and Limberg score assessments—the two key IUS parameters- is well documented and supported by previous study.^23^ For this reason, we excluded patients with a BMI greater than 30 and those with an ileal or colonic stoma, as in these cases reliable image acquisition and interpretation would have been difficult to ensure.

We also found only slight agreement between clinical activity and TH. This limited concordance reflects the well-known discrepancy between symptoms and objective inflammatory burden in CD, highlighting that clinical indices alone are insufficient to capture transmural inflammation. Although the absence of paired endoscopic data represents a limitation, our findings underscore the complementary role of IUS in monitoring disease activity and the need for multimodal assessment.

Based on existing literature, IUS plays a central role in the tight control and monitoring of CD lesions during biological therapy. Our findings further support the utility of IUS as a real-time, accessible, and noninvasive tool that enables timely clinical decision-making directly at the point of care. As more prospective studies emerge, the definitions of complete versus partial TH and their relationship to patient outcomes will continue to evolve, further refining the role of IUS in therapeutic monitoring.

## Supplementary Material

izaf317_Supplementary_Data

## Data Availability

All data are incorporated into the article.
