# Supplementary material for: Early Intestinal Ultrasound Assessment Predicts Therapy Response: An Easy Tool for Clinical Decision-Making
Source: Inflamm Bowel Dis. 2026 Mar 6;32(5):938–44. doi: 10.1093/ibd/izaf317 (PMC13135833; doi:10.1093/ibd/izaf317)
Supplement: izaf317_Supplementary_Data [file izaf317_supplementary_data.zip › supplementary.docx]

Table S1: Dosing regimens and optimization strategies for biologic therapies

| **Biologic** | **Induction regimen** | **Maintenance regimen** | **Dose optimization (if required)** |
| --- | --- | --- | --- |
| **Infliximab** | 5 mg/kg intravenously (IV) at weeks 0, 2, and 6 | 5 mg/kg IV every 8 weeks | 10 mg/kg IV every 8 weeks |
| **Adalimumab** | 160 mg subcutaneously (SC) at week 0, 80 mg SC at week 2 | 40 mg SC every 2 weeks | 40 mg SC every week |
| **Vedolizumab** | 300 mg IV at weeks 0, 2, and 6 | 300 mg IV every 8 weeks | 300 mg IV every 4 weeks |
| **Ustekinumab** | Weight-based IV dose at week 0: 260 mg (<55 kg), 390 mg (55–85 kg), 520 mg (>85 kg) | 90 mg SC every 8 weeks | 90 mg SC every 4 weeks |
| **Risankizumab** | 600 mg IV at weeks 0, 4, and 8 | 360 mg SC every 8 weeks | 360 mg SC every 4 weeks |

Table S2: Rate of ultrasonography response and transmural healing (TH) according to Intestinal Bowel Ultrasound (IBUS) definitions

| **Timepoint** | **IBUS response, n (%)** | **IBUS TH, n (%)** |
| --- | --- | --- |
| 3 months | 40 (28%) | 58 (41%) |
| 12 months | 58 (41%) | 27 (19%) |

Table S3: Agreement between clinical remission, defined as Harvey Bradshaw Index (HBI) <5 and intestinal ultrasound (IUS)-defined transmural healing (TH) at 3 and 12 months. Data are reported as the number of patients in each category, with overall agreement and Cohen’s κ coefficient.

| **Timepoint** | **HBI remission (< 5 points) + TH** | HBI remission + no TH | HBI active (> 5 points) + TH | HBI active (> 5 points) + no TH | Total (n) | Agreement (%) | Cohen’s κ |
| --- | --- | --- | --- | --- | --- | --- | --- |
| Month 3 | 6 | 70 | 2 | 64 | 142 | 49.3% | 0.05 |
| Month 12* | 24 | 72 | 3 | 32 | 131 | 42.7% | 0.10 |

* 11 patients were missing data at Month 12 due to treatment discontinuation.
