# Supplementary figures and images for: Early Intestinal Ultrasound Assessment Predicts Therapy Response: An Easy Tool for Clinical Decision-Making
Source: Inflamm Bowel Dis. 2026 Mar 6;32(5):938–44. doi: 10.1093/ibd/izaf317 (PMC13135833; doi:10.1093/ibd/izaf317)

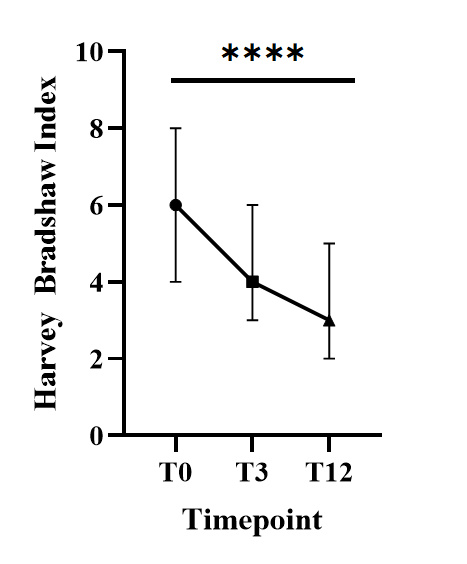

Supplement: izaf317_Supplementary_Data [file izaf317_supplementary_data.zip › Fig S1 . .tiff]
